# Analysis of the detection rate and related factors of thyroid nodules in the healthy population

**DOI:** 10.1515/biol-2025-1079

**Published:** 2025-08-05

**Authors:** Dandan Han, Yu Zhang, Yanyan Zhang, Yongbin Pei, Xiaojing Huang, Lijie Qin, Yayu Zhuo

**Affiliations:** Health Management Center, The First Hospital of Hebei Medical University, Shijiazhuang, 050000, China; Department of Geriatrics, The First Hospital of Hebei Medical University, Shijiazhuang, 050000, China; Department of Ultrasonography, The First Hospital of Hebei Medical University, Shijiazhuang, 050000, China; Department of Neurosurgery, Luquan Hospital, The Second Hospital of Hebei Medical University, No. 501 Huai’an West Road, Shijiazhuang, 050000, China

**Keywords:** thyroid nodules, ultrasonography, logistic regression, metabolic factors, prediction model

## Abstract

In a cross-sectional analysis of 14,973 adults from North China, thyroid nodules (TNs) were detected via high-resolution ultrasonography in 8,104 participants (54.1%), with a higher prevalence among women. The mean age of those with TNs was significantly higher (51.39 ± 15.41 vs 41.83 ± 12.43 years, *p* < 0.001). Univariate analyses indicated that female sex (OR ≈ 2.0), older age (OR ≈ 1.03 per year), elevated low-density lipoprotein cholesterol, low high-density lipoprotein cholesterol, and higher BMI were significantly linked to TNs. In contrast, total cholesterol and uric acid did not reach significance in the final model. A nomogram incorporating these risk factors demonstrated moderate predictive performance (AUC = 0.84 in the training set; 0.78 in the validation set). While the study’s large sample size is a strength, its cross-sectional design limits conclusions about causality, and potential overfitting cannot be excluded. Future research should include thyroid hormone measurements, external validation of the nomogram, and longitudinal follow-up to clarify the role of metabolic factors. These findings highlight the importance of age, sex, and metabolic profiles – particularly dyslipidemia and obesity – in screening for TNs during routine health examinations.

## 1 Introduction

The thyroid is an essential endocrine organ in the human body, and thyroid nodules (TNs) are the most common thyroid disease encountered in clinical practice [1]. The detection rate of TNs can be as high as 67% through palpation or physical examination, aided by high-resolution ultrasound. Approximately 5–15% of patients with TNs may progress to thyroid cancer [2,3,4]. According to the 2018 report by the International Agency for Research on Cancer, the global incidence of thyroid cancer is about 6.7 per 100,000 individuals, with around 190,000 new cases occurring annually in China [5]. Consequently, TNs have emerged as a notable public health concern. Previous studies have associated TNs with iodine intake, genetic predisposition, immune factors, endocrine disturbances, and radiation exposure [6,7,8]. Although several investigations have demonstrated a significant link between TNs and metabolic diseases [9,10,11], these findings remain inconsistent across different populations.

Various risk factors contribute to the occurrence of TNs, including age, gender, obesity, impaired fasting glucose, and insulin resistance [12,13,14]. Moreover, high thyroid stimulating hormone (TSH) levels have been linked to an increased likelihood of malignancy in euthyroid nodules, whereas larger nodules are more often benign [15]. Certain cytomorphological criteria – such as nuclear atypia and the presence of multiple significant parameters – further correlate with a higher risk of malignancy in TNs. Understanding these risk factors is crucial for early detection, prevention, and treatment strategies.

Numerous studies have explored the development of prediction models to improve diagnostic accuracy and clinical decision-making processes. One study proposed a multimodality ultrasound prediction model that integrates conventional ultrasound, shear wave elastography, strain elastography, and contrast-enhanced ultrasound, significantly enhancing the differential diagnosis of TNs ≤ 10 mm [16]. Another investigation employed a Bagged CART model for thyroid cancer prediction, achieving high accuracy, sensitivity, and specificity, with variables such as size, TSH, and blood flow identified as key predictors [17]. Additionally, researchers have examined factors influencing needle visualization during fine-needle aspiration of TNs, leading to the development of a nomogram for predicting the clarity of needle tip display [17,18]. Furthermore, a nomogram model based on clinical and ultrasound features was constructed to optimize thyroid C-TI-RADS classification, demonstrating favorable accuracy and potential clinical utility.

The aim of this study is to determine the prevalence of TNs among a healthy population in North China using high-resolution ultrasonography, while pinpointing key demographic and biochemical risk factors such as cholesterol levels, blood glucose, and uric acid (UA). An important objective is to develop and validate a risk prediction model founded on these factors, thereby enhancing early detection capabilities. Ultimately, the findings are intended to support targeted screening and intervention strategies, contributing to improved prevention and management of TNs, particularly among high-risk groups.

## 2 Methods

This study enrolled 14,973 individuals who underwent routine physical examinations at our hospital’s physical examination center between January 2021 and December 2021. Exclusion criteria included acute or chronic liver and kidney dysfunction, use of thyroid hormone medications within the past 6 months, a history of neck surgery, coexisting malignant tumors, or a history of severe mental and neurological diseases.

### 2.1 Data collection

This study recorded each participant’s height, weight, body mass index (BMI), and blood pressure. Height and weight were measured using a standard height-weight scale, with height recorded in meters (m) and weight in kilograms (kg). BMI was calculated by dividing weight (kg) by the square of height (m). According to the “Guidelines for the Prevention and Control of Overweight and Obesity in Chinese Adults,” a BMI below 18.5 kg/m² was categorized as underweight, 18.5–23.9 kg/m² as normal weight, 24–27.9 kg/m² as overweight, and 28 kg/m² or above as obese.


**Informed consent:** Informed consent has been obtained from all individuals included in this study.
**Ethical approval:** The research related to human use has been complied with all the relevant national regulations, institutional policies, and in accordance with the tenets of the Helsinki Declaration, and has been approved by the Ethics Committee of the First Hospital of Hebei Medical University (S00376).

### 2.2 Laboratory measurements

Venous blood samples were collected from participants after 8–12 h of fasting. Biochemical indicators were measured using an automatic biochemical analyzer (Beckman AU5800). The parameters assessed in this study included fasting blood glucose (FBG), UA, total cholesterol (TC), triglyceride (TG), low-density lipoprotein cholesterol (LDL-C), and high-density lipoprotein cholesterol (HDL-C). According to the “Guidelines for the Prevention and Treatment of Type 2 Diabetes in China (2007 Edition),” the normal range for FBG is 3.9–6.1 mmol/L. The normal reference range for UA is 202.3–416.5 μmol/L. For blood lipids, the reference ranges are TC < 5.2 mmol/L, TG < 2.26 mmol/L, 1.90 mmol/L < LDL-C < 3.10 mmol/L, and 1.00 mmol/L < HDL-C < 1.55 mmol/L. Any abnormality in one or more of these four lipid indicators is defined as dyslipidemia.

### 2.3 Thyroid ultrasonography

Thyroid ultrasonography was performed by qualified ultrasound specialists using a LOGIQE8 color Doppler system equipped with a 3.0–12 MHz probe. Participants lay supine with their shoulders elevated to fully expose the neck. A high-frequency ultrasound probe was then used for multi-sectional scanning to record each nodule’s dimensions (length, width, depth), location, number, echogenicity, margins, and any cystic components. The largest nodule size noted was documented as the TN diameter. Based on these ultrasonographic findings, participants were classified into thyroid nodular status (TNS) and non-TNS groups. Individuals with a history of partial or total thyroidectomy were excluded from the study.

### 2.4 Nomogram construction and evaluation

To develop a predictive model for TNs, the overall cohort was split into two groups: a training set (*N* = 7,500) and a validation set (*N* = 7,473). In the training set, univariate and multivariate logistic stepwise regression analyses were conducted to identify independent predictors of TN. Based on these predictors, a nomogram was constructed. The performance of the resulting nomogram was then evaluated using receiver operating characteristic (ROC) analysis.

### 2.5 Statistical analysis

Data analysis in this study was conducted using SPSS 26.0. Measurement data were expressed as the mean value ± standard deviation (*x̄* ± *s*) and compared using the *t*-test. Categorical data were presented as frequencies (*n* [%]) and compared using the *χ*² test. Additionally, multivariate logistic regression was employed to analyze factors related to the prevalence of TNs, with odds ratios (OR) and corresponding 95% confidence intervals (CI) calculated.

## 3 Results

### 3.1 Comparison of clinical data between the two groups

Table 1 provides a comparative analysis of clinical characteristics between individuals with TNS and those without (Non-TNS). A significant difference in gender distribution was observed: 49.9% of the TNS group were male compared to 58.1% in the Non-TNS group, while 58.9% of the TNS group were female compared to 41.1% in the Non-TNS group (*p* < 0.001). Age also differed notably, with TNS participants averaging 51.39 ± 15.41 years and Non-TNS participants 41.83 ± 12.43 years (*p* < 0.001). By contrast, there was no statistically significant difference in BMI between the two groups.

**Table 1:** Comparison of clinical data between the two group

Variable	TNS group (*n* = 8,104)	Non-TNS group (*n* = 6,869)	*χ*²/*t*	*p*
Gender			122.431	<0.001
Male, *n* (%)	4,007 (49.4%)	4,018 (58.5%)		
Female, *n* (%)	4,097 (50.6%)	2,851 (41.5%)		
Age (years), mean value ± SD	51.39 ± 15.41	41.83 ± 12.43	41.27	<0.001
BMI (kg/m^2^), mean value ± SD	25.48 ± 25.62	24.45 ± 3.77	3.310	0.09

### 3.2 Detection of TNs in different gender and age groups

Table 2 displays the prevalence of TNs by age group and gender, showing both the total number of individuals examined and the corresponding numbers (and percentages) diagnosed with TNs. Overall, 49.93% of males (4,007 out of 8,025) and 58.97% of females (4,097 out of 6,948) were found to have TNs. Additionally, the data indicate that the prevalence of TNs increases with advancing age.

**Table 2:** Comparison of the prevalence of thyroid nodules

Age (year)	Male		Female	
	Total	TN, *n* (%)	Total	TN, *n* (%)
<30	800	290 (36.25%)	838	336 (40.10%)
30–39	2,081	654 (31.43%)	1,920	839 (43.70%)
40–49	1,691	751 (44.41%)	1,472	844 (57.34%)
50–59	1,763	1,019 (57.80%)	1,354	944 (69.72%)
>60	1,690	1,293 (76.51%)	1,364	1,134 (83.14%)
Total	8,025	4,007 (49.93%)	6,948	4,097 (58.97%)

### 3.3 Comparison of glucose and lipid metabolism levels between the two groups

Table 3 compares glucose and lipid metabolism levels between individuals with TNS and those without (Non-TNS). The variables evaluated include total cholesterol (TC), HDL-C, LDL-C, TG, FBG, and UA.

**Table 3:** Comparison of glucose and lipid metabolism levels

Variables	TNS	Non-TNS	*t*	*p*
TC (mM)	5.143 ± 1.093	4.959 ± 1.061	4.775	<0.0001
HDL-C (mM)	1.247 ± 0.2641	1.304 ± 0.2930	12.46	<0.0001
LDL-C (mM)	3.357 ± 0.8007	3.272 ± 0.7750	6.587	<0.0001
TG (mM)	1.668 ± 1.269	1.538 ± 1.478	5.774	<0.0001
FBG (mM)	5.910 ± 1.392	5.569 ± 1.175	41.27	<0.0001
UA (μM)	359.0 ± 97.25	372.4 ± 102.0	3.354	<0.0008

Overall, there were significant differences in all measured parameters between the TNS and Non-TNS groups. TC levels were marginally higher in the TNS group (5.143 ± 1.093 mM) compared with the Non-TNS group (5.059 ± 1.061 mM) (*p* < 0.0001). Both HDL-C and LDL-C also exhibited statistically significant variations between the two groups. Notably, TG and FBG were higher in the TNS group (5.910 ± 1.392 mM and 5.910 ± 1.392 mM, respectively), whereas UA was lower (359.0 ± 97.25 μM in TNS vs 372.4 ± 102.0 μM in Non-TNS).

Since thyroid hormone levels were not evaluated in the participants, we compared key clinical and laboratory parameters between TNS negative (*n* = 57) and TNS positive (*n* = 43) individuals with additional confounder data on iodine intake, radiation exposure, and family history of thyroid disease in a pilot subset (Table S1). The TNS positive group exhibited older mean age (53.4 ± 13.0 vs 46.1 ± 12.4 years, *p* = 0.002), higher TSH (3.0 ± 1.2 vs 2.3 ± 1.0 mIU/L, *p* = 0.01), elevated LDL-C (3.3 ± 0.8 vs 2.8 ± 0.7 mmol/L, *p* = 0.05), and lower HDL-C (1.2 ± 0.3 vs 1.4 ± 0.3 mmol/L, *p* = 0.02) (Table S1). BMI also remained significantly higher in TNS positive individuals (27.0 ± 4.0 vs 24.6 ± 3.1 kg/m², *p* = 0.004) (Table S1).

Family history of thyroid disease was significantly more common in the TNS positive group (20.9% vs 8.8%, *p* = 0.04), while variations in iodine intake and radiation exposure showed no statistical significance in this small sample (Table S1). Taken together, these pilot findings suggest that, beyond metabolic and thyroid function markers, familial predisposition may be relevant in identifying individuals at higher risk for TNs.

### 3.4 Univariate logistic regression analysis of factors related to TNs

Each metabolic or demographic parameter was treated as a continuous variable in the univariate logistic regression analysis. Female sex (compared to male) was associated with approximately double the odds of having TNs (OR = 2.07, 95% CI: 1.88–2.27, Table 4). With each additional year of age, the odds increased by about 3% (OR = 1.03, 95% CI: 1.02–1.04, Table 4). Among the lipid parameters, both higher LDL-C (OR = 1.20, 95% CI: 1.13–1.28) and TC (OR = 1.05, 95% CI: 1.01–1.08) showed positive associations with TNs, whereas HDL-C displayed an inverse relationship (OR = 0.75, 95% CI: 0.68–0.83) (Table 4). Each 1 mmol/L increase in TG raised the odds by 4% (OR = 1.04, 95% CI: 1.02–1.06) (Table 4). Regarding glycemic indicators, higher fasting plasma glucose correlated with a moderate rise in odds (OR = 1.07, 95% CI: 1.03–1.11). Uric acid (scaled per 10 μmol/L) demonstrated a small yet significant effect (OR = 1.01, 95% CI: 1.004–1.016). Finally, each 1 kg/m² increment in BMI resulted in a 6% increase in the likelihood of TNs (OR = 1.06, 95% CI: 1.04–1.08) (Table 4).

**Table 4:** Univariate logistic regression analysis with continuous variables

Variable	*B*	SE	Wald *χ*²	*p*-value	OR (95% CI)
Gender					
(Female = 1, Male = 0)	0.726	0.042	299.195	<0.001	2.07 (1.88–2.27)
Age (per 1-year)	0.029	0.002	196.478	<0.001	1.03 (1.02–1.04)
TC (per 1 mmol/L)	0.045	0.018	6.190	0.013	1.05 (1.01–1.08)
HDL-C (per 1 mmol/L)	-0.292	0.048	37.005	<0.001	0.75 (0.68–0.83)
LDL-C (per 1 mmol/L)	0.182	0.027	45.605	<0.001	1.20 (1.13–1.28)
TG (per 1 mmol/L)	0.039	0.010	14.600	<0.001	1.04 (1.02–1.06)
FBG (per 1 mmol/L)	0.065	0.021	9.558	0.002	1.07 (1.03–1.11)
UA (per 10 μmol/L)	0.011	0.003	12.215	<0.001	1.01 (1.004–1.016)
BMI (per 1 kg/m^2^)	0.054	0.007	57.320	<0.001	1.06 (1.04–1.08)

### 3.5 Multivariate logistic regression analysis of factors related to the occurrence of TNS

Each metabolic and demographic parameter is entered into a multivariate logistic regression model, controlling for all other covariates. Female sex remains significantly associated with a nearly twofold odds of having TNs (OR = 1.97, 95% CI: 1.80–2.14), while each additional year of age confers a 3% increment in odds (OR = 1.03, 95% CI: 1.02–1.04) (Table 5). Notably, LDL-C retains a strong positive association (OR = 1.18, 95% CI: 1.11–1.27), whereas HDL-C is inversely related (OR = 0.81, 95% CI: 0.74–0.89) (Table 5). In contrast, TC is no longer statistically significant in this adjusted model (*p* = 0.092). Elevated TG, FBG, and increased UA each display modest but significant contributions to nodule risk. BMI (OR = 1.05, 95% CI: 1.03–1.06 per 1 kg/m^2^ increment) also remains an independent predictor of TNs (Table 5).

**Table 5:** Multivariate logistic regression analysis with continuous variables

Variable	*B*	SE	Wald *χ*²	*p*-Value	OR (95% CI)
Gender					
(Female = 1, Male = 0)	0.680	0.047	208.674	<0.001	1.97 (1.80–2.14)
Age (per 1-year)	0.028	0.002	171.202	<0.001	1.03 (1.02–1.04)
TC (per 1 mmol/L)	0.032	0.019	2.843	0.092	1.03 (0.99–1.08)
HDL-C (per 1 mmol/L)	−0.214	0.049	19.150	<0.001	0.81 (0.74–0.89)
LDL-C (per 1 mmol/L)	0.165	0.028	34.732	<0.001	1.18 (1.11–1.27)
TG (per 1 mmol/L)	0.020	0.010	4.200	0.040	1.02 (1.00–1.05)
FBG (per 1 mmol/L)	0.046	0.020	5.290	0.021	1.05 (1.01–1.10)
UA (per 10 μmol/L)	0.007	0.003	5.444	0.020	1.01 (1.00–1.01)
BMI (per 1 kg/m²)	0.045	0.008	31.641	<0.001	1.05 (1.03–1.06)

### 3.6 Construction of a prediction model for the occurrence of TNs

**Figure 1:**
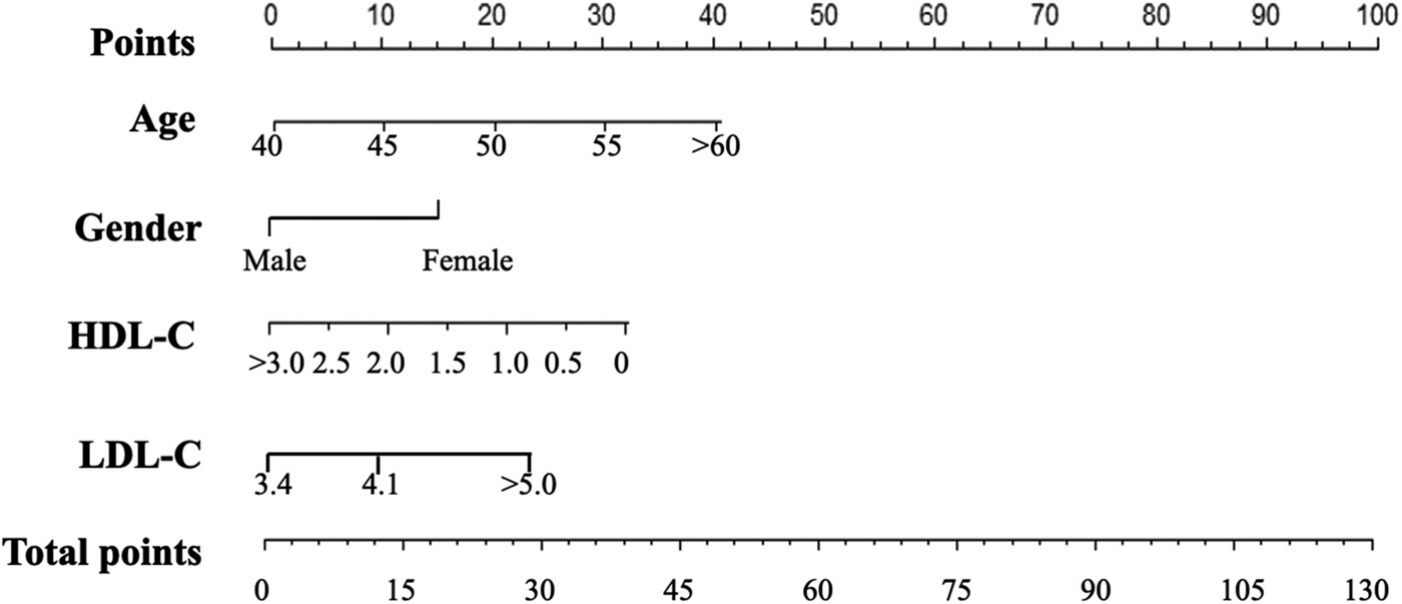
Nomogram model for the prevalence of thyroid nodules.

**Figure 2:**
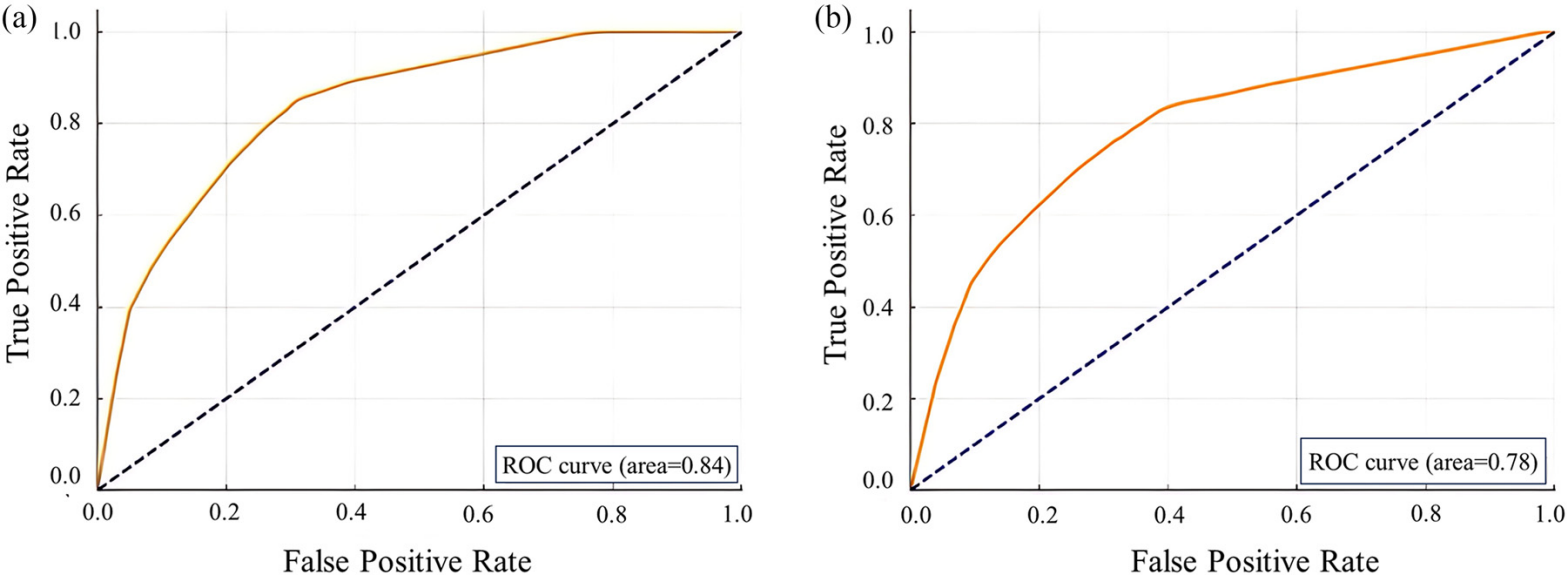
ROC curve for the training (a) and validation set (b).

Based on the multivariate logistic regression findings, a nomogram was constructed to predict the occurrence of TNs (Figure 1). The participants were stratified into low risk (points < 30), intermediate risk (30–80 points), and high risk (over 80 points). In the training set, the model achieved an area under the receiver operating characteristic curve (AUC) of 0.84 (95% CI: 0.83–0.85), while in the validation set, the AUC was 0.78 (95% CI: 0.77–0.79) (Figure 2a and b).

## 4 Discussion

Among the 14,973 participants assessed, the newly confirmed prevalence of TNs was 54.1%. Notably, this prevalence was higher among women and older adults. In particular, the mean age was significantly higher in the TN group relative to those without TNs, and female participants demonstrated an increased risk compared to their male counterparts. Furthermore, TNs were significantly linked to several metabolic factors. Individuals with TNs exhibited elevated levels of LDL-C, lower levels of HDL-C, higher BMI, and increased blood glucose, underscoring a strong association between TN presence and adverse metabolic profiles.

The prevalence of TNs in our study (54.1%) aligns with the previously reported range of 20–68%, influenced by regional factors such as iodine intake and dietary habits. A large-scale study in Southwest China with over 120,000 participants found a similar prevalence [19]. Furthermore, our findings that TNs are more common in women and older adults are consistent with prior epidemiological research [20]. Regarding metabolic risk factors, our study found strong associations between TNs and higher LDL-C, lower HDL-C, increased fasting glucose levels, and BMI. These results are in agreement with prior study, which reported significant metabolic abnormalities among TN patients [21]. Additionally, studies have confirmed that metabolic syndrome components, particularly impaired glucose metabolism, increase TN prevalence [22] and that proper glycemic control may help reduce TN risk [23]. Overall, our results reinforce the growing evidence linking metabolic disorders, particularly dyslipidemia and obesity, to TN development.

Our predictive model for TN risk demonstrated moderate accuracy, with an AUC of 0.84 in the training set and 0.78 in the validation set, suggesting a potential overfitting issue. Overfitting is a common limitation in predictive models, particularly when the number of predictors exceeds the available sample size, which may reduce the model’s generalizability to new populations [24]. Additionally, defining “high-risk” populations remains challenging due to the high prevalence of metabolic disorders such as obesity and hyperlipidemia, which complicates the establishment of a clear cut-off for risk stratification. Previous studies have highlighted the difficulty of determining optimal thresholds, as misclassification can lead to unnecessary interventions or missed diagnoses [25]. Our model currently provides a probability estimate rather than a strict classification, and further refinement of cut-off points through external validation is essential. To improve robustness, future research should focus on prospective or multi-center studies with diverse cohorts to better assess model performance and avoid selection bias [26]. Moreover, the integration of machine learning techniques and nested cross-validation approaches has been suggested as a way to enhance model accuracy while reducing overfitting risks [27]. Ultimately, before widespread clinical adoption, further refinements and validations are necessary to establish more reliable predictive thresholds for TN risk.

Thyroid ultrasound remains the gold standard for detecting TNs; however, routine screening of large populations may not be clinically necessary or cost-effective without additional refinement. High-resolution ultrasound has significantly improved the detection of TNs, but its high sensitivity can lead to the identification of small, asymptomatic nodules, raising concerns about overdiagnosis and unnecessary interventions. Studies have shown that the use of US-based risk stratification systems, such as ACR TI-RADS and the Chinese Thyroid Imaging Reporting and Data System (C-TIRADS), aims to reduce unnecessary biopsies while maintaining diagnostic accuracy [28,29]. However, despite these advancements, overdiagnosis remains a challenge, particularly in cases where clinically insignificant nodules are detected and treated aggressively [30]. Overdiagnosis inflates prevalence estimates and may lead to unnecessary fine-needle aspiration biopsies or even surgeries. A systematic review found that the unnecessary biopsy rate varies significantly across different stratification systems, with ACR TI-RADS demonstrating relatively lower sensitivity but better specificity in avoiding overtreatment [31]. Targeted screening strategies should be explored to identify individuals at the highest risk based on metabolic and hormonal profiles [30]. Combining radiomics with ultrasound-based risk stratification systems has been explored as a means to improve specificity and reduce false-positive cases [32]. To mitigate unnecessary interventions, risk stratification methods such as elastography, which assesses tissue stiffness, and additional biomarkers may be crucial in distinguishing benign from malignant nodules more effectively [33]. Integrating metabolic and hormonal data with our nomogram could enhance risk stratification. Prior research suggests that combining ultrasound features, thyroid function tests, and molecular markers can improve specificity and reduce unnecessary biopsies [34]. Additionally, metabolomic profiling has emerged as a promising tool for distinguishing benign from malignant nodules and could be incorporated into future screening models [35]. To better understand the natural history of TNs, prospective or longitudinal follow-up studies are needed to track how nodules evolve over time in individuals with different metabolic profiles. Such studies could clarify which factors truly drive nodule progression vs those associated with stable, benign lesions, ultimately refining screening guidelines and treatment strategies [36].

Our study did not include detailed data on dietary intake, physical activity, radiation exposure, or socioeconomic status, which are all potential confounding factors influencing the development of TNs. Prior research suggests that iodine intake and dietary goitrogens significantly impact thyroid health, with both iodine deficiency and excess being associated with TN risk [37,38]. Additionally, physical inactivity and obesity have been linked to increased TN prevalence, emphasizing the role of metabolic health in thyroid disorders [39]. Socioeconomic status has also been identified as a factor influencing TN risk, as lower socioeconomic status is often associated with poorer diet, reduced access to healthcare, and increased environmental exposure to endocrine-disrupting chemicals [40]. Furthermore, environmental pollutants, including radiation exposure and industrial chemicals, have been implicated in thyroid carcinogenesis [41]. To enhance our understanding of these relationships, future research should incorporate validated lifestyle questionnaires, urinary iodine concentration measurements, and detailed radiation exposure history to clarify the interplay between lifestyle, environmental, and metabolic factors in TN pathogenesis.

One limitation of our study is the absence of uniform thyroid function test data, including TSH, FT4, and FT3 levels. Without these biomarkers, it is difficult to discern whether subclinical hypothyroidism or hyperthyroidism may have influenced the metabolic factors associated with TNs. Previous research has emphasized the importance of thyroid hormone measurements in TN studies, as variations in TSH levels have been linked to both nodule formation and malignancy risk [42]. Furthermore, the lack of thyroid function data limits our ability to distinguish whether metabolic alterations observed in TN patients are due to intrinsic thyroid dysfunction or other confounding variables [30]. This gap in data also affects the interpretation of our findings, as some studies suggest that missing hormone assessments can lead to potential misclassification of TN risk factors and incomplete metabolic profiling [43]. Given these constraints, future studies should incorporate comprehensive thyroid function testing to better delineate the relationship between thyroid hormones and metabolic risk factors in TN development.

Our findings highlight the potential clinical value of integrating metabolic indicators into routine evaluation of TNs detected by ultrasound. Incorporating thyroid hormone levels, lifestyle factors, and advanced imaging or biomarker techniques will help guide more precise risk stratification and more effective, patient-centered management of TNs, ultimately reducing unnecessary procedures and focusing resources on patients most likely to benefit.

## Supplementary Material

Supplementary Table
