# Supplementary material for: Analysis of the detection rate and related factors of thyroid nodules in the healthy population
Source: Open Life Sci. 2025 Aug 5;20(1):20251079. doi: 10.1515/biol-2025-1079 (PMC12326299; doi:10.1515/biol-2025-1079)
Supplement: Supplementary Table [file biol-2025-1079-sm.pdf]

Supplementary material

Table S1: Key clinical, laboratory, and potential confounder variables

Variable	TNS negative (*n* = 57)	TNS positive (*n* = 43)	*p*-value
Age (years)	46.1 ± 12.4	53.4 ± 13.0	0.002
Sex			0.40
Male, *n* (%)	27 (47.4%)	17 (39.5%)	
Female, *n* (%)	30 (52.6%)	26 (60.5%)	
TSH (mIU/L)	2.3 ± 1.0	3.0 ± 1.2	0.01
FT3 (pmol/L)	4.1 ± 0.7	4.5 ± 1.1	0.03
FT4 (pmol/L)	14.7 ± 2.4	15.7 ± 2.6	0.08
LDL-C (mmol/L)	2.8 ± 0.7	3.3 ± 0.8	0.05
HDL-C (mmol/L)	1.4 ± 0.3	1.2 ± 0.3	0.02
FPG (mmol/L)	5.4 ± 0.8	5.8 ± 1.2	0.10
BMI (kg/m^2)	24.6 ± 3.1	27.0 ± 4.0	0.004
UA (μmol/L)	354 ± 88	379 ± 82	0.20
Iodine Intake, *n* (%)			0.20
Low (<100 μg/day)	4 (7.0%)	6 (14.0%)	
Adequate (100–300 μg/day)	43 (75.4%)	26 (60.5%)	
High (>300 μg/day)	10 (17.5%)	11 (25.6%)	
Radiation exposure, *n* (%)			0.30
None	48 (84.2%)	33 (76.7%)	
Medical (e.g., prior therapy)	7 (12.3%)	9 (20.9%)	
Occupational/Other	2 (3.5%)	1 (2.3%)	
Family history of thyroid disease, *n* (%)			0.04
Yes	5 (8.8%)	9 (20.9%)	
No	52 (91.2%)	34 (79.1%)	
